# Comparison of Three Eccentric Overload Training Strategies on Power Output and Interlimb Asymmetry in Youth Soccer Players

**DOI:** 10.3390/ijerph18168270

**Published:** 2021-08-04

**Authors:** Alejandro Moreno-Azze, José Luis Arjol-Serrano, David Falcón-Miguel, Chris Bishop, Oliver Gonzalo-Skok

**Affiliations:** 1 Faculty of Health Sciences, University of San Jorge, 50830 Zaragoza, Spain; jlarjol@usj.es; 2 Faculty of Health & Sport Sciences, University of Zaragoza, 22001 Huesca, Spain; dfalcon@unizar.es; 3 London Sport Institute, Faculty of Science and Technology, Middlesex University, London NW4 4BT, UK; c.bishop@mdx.ac.uk; 4 Return to Play Department, 41005 Sevilla, Spain; oligons@hotmail.com

**Keywords:** eccentric training, resistance training, injury prevention

## Abstract

Background: The present study compared the effects of performing the lateral squat exercise in three different formats from eccentric overload training on concentric/eccentric peak/mean power and inter-limb asymmetries in young soccer players. Methods: Forty-five young male (U-17) soccer players were distributed into three groups. Two groups performed the same training volume with both legs, beginning with the weaker leg (SVW, *n* = 15) or with the stronger leg (SVS, *n* = 15). The third group executed double volume with the weaker leg and also commenced with such leg (DVW, *n* = 15) in the lateral squat during a 10-week period. Pre- and post-intervention metrics included concentric and eccentric peak/mean power during the lateral squat test and their corresponding asymmetries. Results: All groups improved all power variables. Concentric mean and peak power asymmetry were substantially reduced in the SVW (ES: 0.89), DVW (ES: 0.43), and in SVW (ES: 1.60). Eccentric mean and peak power asymmetry were also substantially decreased in SVW (ES: 0.81) and in DVW (ES: 0.68). Between-group analyses showed substantially better performance in concentric and eccentric variables with stronger and weaker legs in SVW and DVW groups compared with SVS. Conclusions: Those groups which started with the weaker leg showed greater both power enhancements and reductions on inter-limb asymmetries.

## 1. Introduction

Soccer players are continuously performing high-intensity actions, such as jumps, accelerations, decelerations, and changes of direction [1]. However, these skill executions often occur in different volumes between one leg and the other [2]. Therefore, between-limb asymmetries research is of increasing relevance nowadays in many sports [3,4]. Asymmetries are defined as the difference in performance or function of one leg relative to the other [5]. Players with asymmetries of over 15% seem to have higher risk of lower extremity injury in comparison with scores below this threshold [6]. On the other hand, asymmetries that are <10% are suggested as a possible threshold to aim for when aiming for return to training or competition [7,8].

After a competitive soccer game, players show muscle damage with homeostatic balance is often not restored until after 72 h [9]. Players involved in congested schedules frequently compete twice per week; they are often not fully ready to compete at their best. Injury rates have been shown to significantly increase when compared to playing a single game per week [10]. On the other hand, it has been shown that stronger players exhibit reduced creatine kinase, a marker of muscle damage, 48 h post game [11].

Historically, training programs have been developed from vertical bilateral isokinetic movements [12,13,14] to multidirectional, unilateral, eccentric overload movements, suggesting improvements in acceleration, sprinting, jumping, and change of direction (COD) [15,16,17]. Despite of this important progress, there is still not enough information about the assessment of muscle power during resistance training [18].

In an attempt to reduce injury risks, eccentric overload training (EOT) has been implemented as a training strategy so that players can improve strength and subsequently withstand cumulative stress experienced from competition and training [19,20]. When EOT is executed 24 to 48 h after a game, it has been shown to avoid physical performance reduction in subsequent matches [19]. Therefore, it seems that EOT should be considered as a viable training strategy for soccer players to enhance physical readiness for competition.

In recent years, the inclusion of training programs focusing on eccentric overload has exponentially increased in scientific literature. Mostly, the effects of such training programs have been analyzed on specific physical abilities, such as jumping, sprinting, or changing direction [15,20,21,22,23,24]. Nevertheless, even though mean and peak power have been examined, information concerning the influence of EOT on these variables is scarce [25,26,27,28]. Naturally, as they perform one task over time, one would expect them to improve with practice. However, peak and mean power (from EOT) have been shown to be substantially improved after performing several sets of half-squats and lunges after a period of 6–9 weeks [25,27,28]. Some EOT interventions have been developed with the use of a control group [25], between unilateral or bilateral exercises, or comparing variations between two different inertial loads [26]. However, to the authors’ knowledge, no information currently exists about the effects of performing different training strategies, such as training volume or the leg used to start the intervention while employing the same exercise on power measurements through the use of rotational devices.

Whilst EOT interventions have been conducted to determine the effects on measures of strength, far less training studies have been conducted to determine the effects on inter-limb asymmetry in team-sports athletes [20,21,23,29,30]. Specifically, there are only two studies [21,23] that have analyzed the effect of EOT on inter-limb asymmetries, both of which showed significant reductions in jumping asymmetry. For example, young soccer players showed reductions in asymmetry from single-leg CMJ after unilateral EOT training, starting with the weaker leg. Additionally, triple hop tests’ asymmetry reduction were shown in a group that performed a double volume in the weaker leg in an unilateral EOT training, beginning with the weaker leg [21]. Furthermore, greater training effects have been shown during change of direction tasks, jump performance, handball throwing velocity, and jumping asymmetry in a group that performed an EOT intervention compared to a resistance-cable training intervention [23]. Despite these findings, there is no study analyzing the effects of EOT on power inter-limb asymmetries measurements (i.e., peak or mean power). Therefore, the main aims of the current study were: (1) to compare the effects of performing different unilateral EOT interventions on concentric and eccentric peak and mean power measured through the lateral squat and (2) to examine the training intervention effects on inter-limb asymmetry in young soccer players. The authors hypothesized that all EOT interventions would be effective at improving concentric and eccentric peak and mean power and reducing inter-limb asymmetry. It is of note that starting the training program with the weaker leg and performing double volume would be more effective than beginning with the stronger leg or performing the same volume in both legs.

## 2. Materials and Methods

### 2.1. Experimental Approach to the Problem

Subjects were distributed into three unilateral eccentric overload groups based on their mean concentric power performance, by means of a randomized study design (A-B-C). The routine in the first group was executing the same training volume with both legs, beginning with the weaker leg (SVW, *n* = 15). The second group executed the double training volume with the weaker leg and also commenced with such leg (DVW, *n* = 15), and the third group implemented the same training volume with both legs, starting with the stronger leg (SVS, *n* = 15). The leg that scored the lowest value of the two sides in the pre-test was denoted as the weaker leg. One week before carrying out the reliability analysis, one familiarization session was done. As at least three sessions are needed to stabilize eccentric overload power measurements [26], the reliability analysis was performed with three testing sessions in the lateral squat test three weeks before the training period (1 per week). This test was carried out one week after the training period to examine the training effects.

### 2.2. Subjects

Forty-five young (U-17) male soccer players (age: 15.6 ± 1.0 y, height: 173.9 ± 6.8 cm, body mass: 63.7 ± 8.2 kg) belonging to a Spanish second division professional soccer club academy participated in the study voluntarily. Using G*Power 3.1 software (University of Düsseldorf, Düsseldorf, Germany), it was determined that 34 subjects were needed in order for the study to have a statistical power of 0.80 with an alpha level of 0.05 and an effect size of 0.5 [31]. Data collection took place between the seventh and ninth month of the season. This nine-month period was divided into 2 pre-season month periods and 7 competitive month periods. These players had ~9 h programmed training based on combining soccer (4 sessions) and strength/power (1 session) sessions plus one competitive match per week. The subjects’ strength training experience was 1.80 ± 0.72 years (range: 1 to 3 years). Written informed consent was obtained from both the players and their legal guardians before beginning the investigation. The current study was approved by the institutional research ethics committee and conformed to the recommendations of the Declaration of Helsinki. It should be noted that the sample that participated in the present study is the same as the sample of a previous study [21].

### 2.3. Procedures

#### 2.3.1. Training Intervention

During 10 consecutive weeks, in addition to their normal soccer training, participants performed one EOT session per week. These sessions (Table 1) were performed on Tuesday or Wednesday, 48 h after the last match, and at least 48 h before the next match. The training intervention (Table 1) consisted of 2 sets of 6–10 repetitions of lateral squat using a portable conical pulley (Versapulley, Costa Mesa, CA, USA; inertia 0.27 kg/m^2^, speed: force ratio (i.e., as the ratio increases, the training intensity also increases) 1–3 out of 4 [32], and transmission pulley/harness was setup from the hip of the working leg) after a standardized warm-up (i.e., 5 min jogging, dynamic stretches, and 2 sets of lateral squats with each leg for 8 repetitions, doing the last 3 repetitions as fast as possible). Players who did not complete at least 80% of the training sessions were excluded from the analysis. Given that many injury mechanisms often occur in the frontal plane, strength performance out of the sagittal plane was considered appropriate, reproducing frequently multi-directional movement patterns [33]. Subjects were verbally encouraged before and during the exercise, if needed, to perform the concentric phase as fast as possible while delaying the breaking action to the last third of the eccentric phase. Between-legs and sets recovery were 30 s and 3 min, respectively. It is worth mentioning that the present study performed a training intervention carried out in a previous study [21]. The main researcher, together with two experienced S&C coaches, controlled every training session, providing verbal encouragement to each participant.

#### 2.3.2. Lateral Squat Test

**Figure 1:**
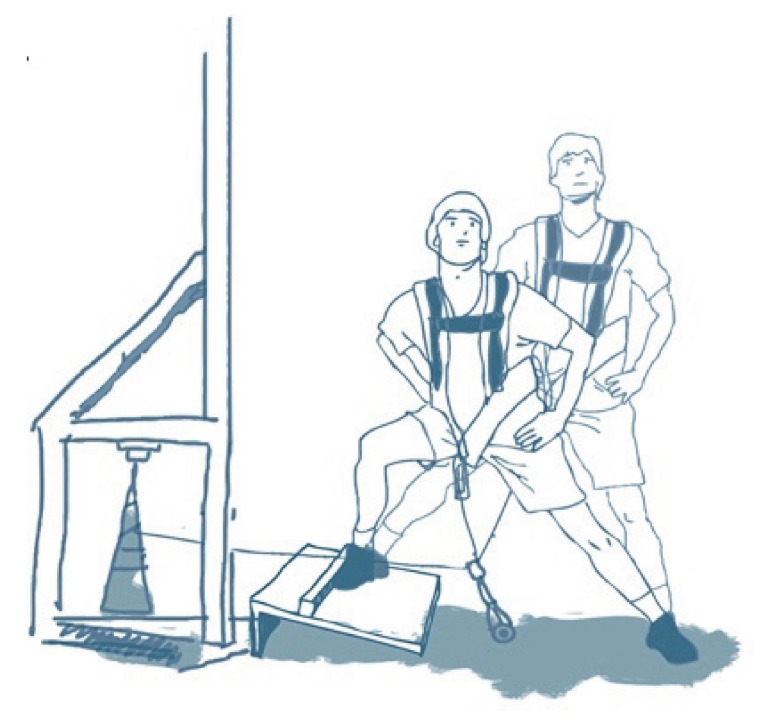
Eccentric overload variable unilateral exercise and the corresponding force-vector application: lateral squat (mediolateral/lateromedial).

The lateral squat test (Figure 1) was carried out 72 h before starting the training intervention. They were asked not to perform intense exercise on the day before the scheduled test time. This test consisted of performing 10 repetitions. If there was a double consecutive warning of the 10% decrement of the first three repetitions’ mean power, they had to stop as well. The lateral squat test depth was standardized, delaying the breaking action to the last third of the eccentric phase. Before performing the exercise, the subjects’ knee angle was measured forming a 90° angle and marked with tape on a vertical pole on a firm base. The pole was positioned next to the subject when performing the test. One attempt was allowed with each leg. When the rule was not respected, the repetition was not considered valid. Before starting the power assessment, a standardized warm-up (i.e., 5 min jogging, dynamic stretching, 10 bilateral squats, core exercises, 10 unilateral squats) was executed. Players were required to begin this unilateral test with the left leg through a conical pulley (Versapulley, Costa Mesa, CA, USA; inertia 0.27 kg/m^2^, speed:force ratio (i.e., as the ratio increases, the training intensity also increases) 1 out of 4, and transmission pulley/harness was setup from the hip of the working leg). Mean concentric power (ConMean), mean eccentric power (EccMean), maximum peak concentric power (ConPeak), and maximum peak eccentric power (EccPeak) were recorded through a rotational encoder and its specific software (SmartCoach v.5.6.0.8, SmartCoach Europe AB, Stockholm, Sweden). Between-legs and sets recovery were 1 min and 3 min, respectively. Data analysis was performed using the mean of the best 3 concentric repetitions from the best set (i.e., greater mean concentric power) of each leg, obtaining results for stronger and weaker leg. Thus, the final variables analyzed were mean concentric power stronger leg (ConMean stronger), mean concentric power weaker leg (ConMean weaker), mean eccentric power stronger leg (EccMean stronger), mean eccentric power weaker leg (EccMean weaker), maximum peak concentric power stronger leg (ConPeak stronger), maximum peak concentric power weaker leg (ConPeak weaker), maximum peak eccentric power stronger leg (EccPeak stronger), and maximum peak eccentric power weaker leg (EccPeak weaker).

### 2.4. Statistical Analyses

Assessed data are presented as mean ± standard deviation (SD). To analyze the normally distributed data, the Shapiro–Wilk test was used. For within group comparisons, paired t-test were applied to detect significant differences, established a priori at *p* < 0.05 in any variable. Before analyzing, avoiding any bias from a non-uniformity error, all data were log-transformed. A two-way random intraclass correlation coefficient (ICC) with absolute agreement and 90% confidence interval in addition to the coefficient of variation (CV) was used to analyze the between-session reliability. Previous research of Koo and Li (2016) was used to interpret ICC values [34], where >0.9 = excellent, 0.75–0.9 = good, 0.5–0.75 = moderate, and <0.5 = poor, and CV values were considered acceptable if <10% [35]. Pooled pre-training SD in the selective variables was used to determine the effect size (ES, 90% CI). Cohen ES statistics threshold values were >0.2 (small), >0.6 (moderate), and >1.2 (large) [36]. Chances that the differences in performance were better/greater similar or worse/smaller were calculated for within and between-group comparisons. Quantitative chances of beneficial/better or detrimental/poorer effect were assessed qualitatively as follows: <1%, most likely not; >1–5%, very unlikely; >5–25%, unlikely; >25–75%, possible; >75–95%, likely; >95–99%, very likely; and >99%, most likely [36]. If the chance that the true value is >25% beneficial and >0.5% chance that it is harmful, the clinical effect was considered as unclear.

Likewise, if the odds ratio of benefit/harm was <66, it continued being unclear. In the same way, when odds ratio of benefit/harm was >66, the clinical inference was declared as beneficial. Statistical analysis was performed with SPSS for MAC (Version 25.0; SPSS Inc., Chicago, IL, USA) and two specific Excel spreadsheets from sportsci.org were used to examine both the between-group (xCompare2groups.xls) and within-group (xPostOnlyCrossover.xls) comparisons.

Based on current recommendations [37], inter-limb asymmetries were calculated with the following formula:

100/Max Value (right and left) × Min Value (right and left) × −1 + 100.3.

## 3. Results

Ten players were excluded from the analysis because they were transferred to another team or due to changes made in their training schedule or not completing 80% of the training sessions. No injuries were registered during the training program. Thus, 35 players (15.4 ± 0.7 years, 174.9 ± 5.8 cm, 64.2 ± 7.0 kg) were included in the subsequent data analysis. Finally, the final sample size was 10 players for SVW, 11 for DVW, and 14 for SVS. Attending to these dropouts, no significant differences were found between groups at baseline. Attending to performance (the strongest leg from the mean concentric power data test was defined as the strongest leg), seven players showed a greater performance with the right leg and three with the left leg in the SVW, five (right) and six (left) in the DVW, and nine (right) and five (left) in the SVS.

Assessed mean values and reliability data are presented in Table 2. CV values were >10% (range 16.28% to 24.87%), and all tests showed between a good and moderate reliability (ICC = 0.66 to 0.84) (Table 2). Regarding within-group changes, all groups substantially improved all power variables with right, left, stronger, and weaker leg (ES = 0.77 to 2.24). In reference to asymmetries, SVW asymmetry improvements were found in ConMean (ES = 0.89), EccMean (ES = 0.81), and ConPeak (ES = 1.60), DVW asymmetry enhancements were shown in ConMean (ES = 0.43) and EccPeak (ES = 0.68), and SVS better results were found in EccMean (ES = 0.63) (Table 3).

**Figure 2:**
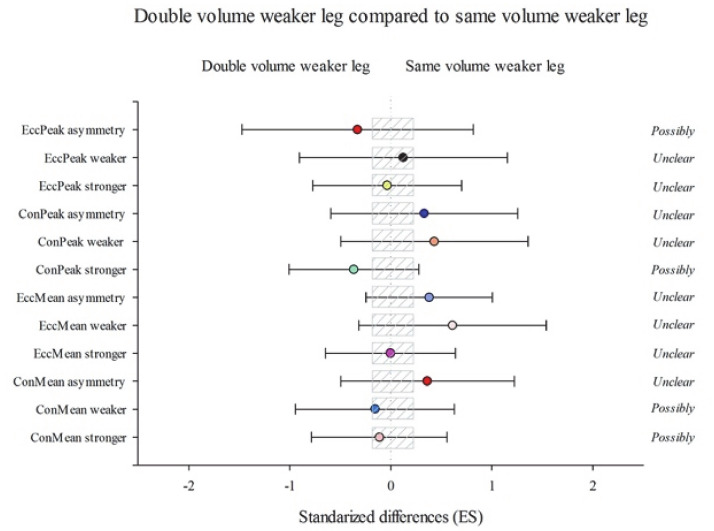
Efficiency of the unilateral eccentric overload training performing the double volume with the weaker leg starting with the weaker leg (DVW) compared with the unilateral eccentric overload training performing the same volume with both legs, starting with the weaker leg (SVW). Training program to improve a lateral squat in mean concentric power (ConMean) with the stronger and the weaker leg and the corresponding asymmetry; mean eccentric power (EccMean) with the stronger and the weaker leg and the corresponding asymmetry; maximum peak concentric power (ConPeak) with the stronger and the weaker leg and the corresponding asymmetry; maximum peak eccentric power (EccPeak), with the stronger and the weaker leg and the corresponding asymmetry. (Bars indicate uncertainty in the true mean changes with 90% confidence limits.) Trivial areas were the smallest worthwhile change (see Section 2).

**Figure 3:**
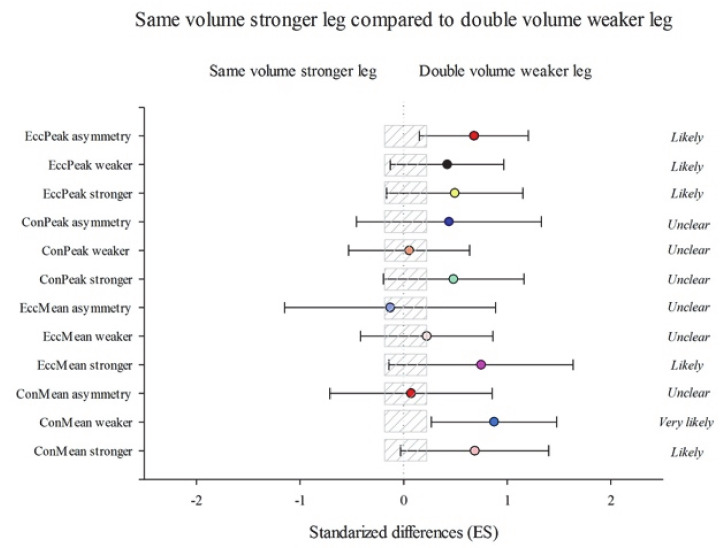
Efficiency of the unilateral eccentric overload training performing the same volume with both legs starting with the stronger leg (SVS) compared with the unilateral eccentric overload training performing the double volume with the weaker leg, starting with the weaker leg (DVW). Training program to improve a lateral squat in mean concentric power (ConMean) with the stronger and the weaker leg and the corresponding asymmetry; mean eccentric power (EccMean) with the stronger and the weaker leg and the corresponding asymmetry; maximum peak concentric power (ConPeak) the stronger and the weaker leg and the corresponding asymmetry; maximum peak eccentric power (EccPeak) with the stronger and the weaker leg and the corresponding asymmetry. (Bars indicate uncertainty in the true mean changes with 90% confidence limits.) Trivial areas were the smallest worthwhile change (see Section 2).

**Figure 4:**
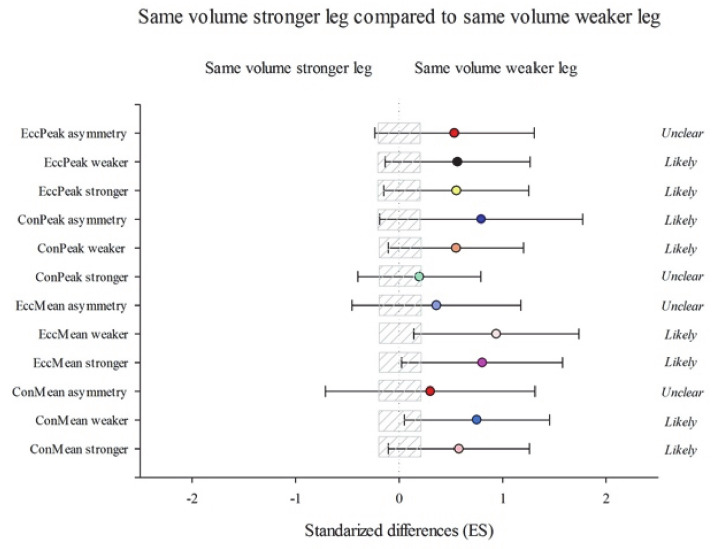
Efficiency of the unilateral eccentric overload training performing the same volume with both leg starting with the stronger leg (SVS) compared with the unilateral eccentric overload training performing the same volume with both legs starting with the weaker leg (SVW). Training program to improve a lateral squat in mean concentric power (ConMean) with the stronger and the weaker leg and the corresponding asymmetry; mean eccentric power (EccMean) with the stronger and the weaker leg and the corresponding asymmetry; maximum peak concentric power (ConPeak) the stronger and the weaker leg and the corresponding asymmetry; maximum peak eccentric power (EccPeak) with the stronger and the weaker leg and the corresponding asymmetry. (Bars indicate uncertainty in the true mean changes with 90% confidence limits.) Trivial areas were the smallest worthwhile change (see Section 2).

According to between-group changes, group DVW showed substantial improvements in ConMean stronger (2.83% (−18.02; 15.17); *p* = 1.00) ConMean weaker (3.34% (−18.57; 14.74); *p* = 1.00), ConPeak stronger (9.47% (−24.02; 7.86); *p* = 0.98), and EccPeak asymmetry (20.74% (−64.65; 77.75); *p* = 1.00) than SVW group (Figure 2). Likewise, group DVW performed substantial improvements as opposed to the SVS group in ConMean stronger (16.27% (−0.64; 36.06); *p* = 0.40) ConMean weaker (23.77% (6.73; 43.53); *p* = 0.16), EccMean stronger (13.02% (−2.29; 30.73); *p* = 0.39), EccPeak stronger (15.87% (−4.86; 41.13); *p* = 0.50), EccPeak weaker (13.35% (−3.77; 33.52); *p* = 1.00), and EccPeak asymmetry (113.57% (18.64; 284.45); *p* = 1.00) (Figure 3). On the other hand, group SVW obtained more substantial improvements than the SVS group in ConMean stronger (13.56% (−2.27; 31.96); *p* = 0.27) ConMean weaker (20.18% (1.25; 42.66); *p* = 0.32), EccMean stronger (14.05% (0.42; 29.52); *p* = 0.31), EccMean weaker (18.59% (2.6; 37.08); *p* = 0.10), ConPeak weaker (15.51% (−2.72; 37.17); *p* = 0.67), ConPeak asymmetry (76.54% (−12–61; 256.63); *p* = 1.00), EccPeak stronger (17.97% (−4.36; 45.52); *p* = 0.51), and EccPeak weaker (18.39% (−3.97; 45.96); *p* = 0.36) (Figure 4).

## 4. Discussion

The aim of the current study was to compare the effects of performing different unilateral strength-training interventions on concentric and eccentric peak and mean power and explore their corresponding effects on inter-limb asymmetries. The main findings were as follows: (1) mean and peak power performance were very likely to most likely improved in all groups, (2) a moderate reduction in mean and maximum peak concentric and eccentric asymmetry was achieved in those groups that started the program with the weaker leg, and (3) the double volume starting with the weaker leg group developed better results in ConMean compared to SVS and SVW.

Substantial improvements were found in ConMean (ES: 1.16 to 2.24), which is comparable with the range reported in previous studies (ES: 0.85 to 0.91) [25,28]. Regarding ConPeak power, other research studies [27] showed lower training effects (ES: 0.38 to 0.84) in comparison to the current study (ES: 0.77 to 1.63). Due to the fact that these research studies had different training strategies (e.g., duration, training programmes conducted, acute variables programmed), it is challenging to determine the truly most effective aspect of such programmes for the improvement of this metric. Nevertheless, training for 10 weeks (as opposed to 5–7 weeks) with a lower number of sets and progressive repetitions (2 sets × 12 to 20 reps instead of 2 to 4 sets × 12 to 48 reps) seems to show better improvements. Therefore, despite more research being needed, volume and progression appear to be key variables to consider for improvements in concentric power output in iso-inertial pulley training. None of the previous studies recently mentioned have shown between-leg dominance (i.e., right or left) nor between-leg performance (i.e., stronger or weaker); thus, direct comparisons are not possible. One study analyzed the effects of performing different EOT interventions, differing on the leg used to start the intervention, showing better ES results in triple hop (ES: 0.51 to 0.71 vs. 0.00 to 0.15) and in bilateral CMJ jump (ES: 0.48 vs. 0.30) in those groups that started their training with the weaker leg [21]. Supporting the research study recommendation already mentioned, training effects were considerably greater in concentric power in those groups that started with the weaker leg (ES: 1.07 to 2.24 in ConMean and 0.77 to 1.63 in ConPeak) compared to SVS (ES: 1.16 to 1.53 in ConMean and 1.25 to 1.33 in ConPeak) in the present study. It should be noted that, even though CV values suggested a poor consistency in concentric power test (16.28–19.46%), training effects were much greater (ConMean stronger: 49.89–70.94%, ConMean weaker: 78.88–113.80%, ConPeak stronger: 51.29–57.31%, and ConPeak weaker: 47.48–89.61%) in all variables, thus inspiring confidence that true change was apparent as a consequence of the training interventions. When considering between-group differences, those groups that started with the weaker leg compared to SVS showed greater enhancements in ConMean and ConPeak for SVW and in ConMean for DVW. However, DVW achieved better results in the ConMean, while ConPeak results were unclear compared to SVW. Thus, this finding suggests that adding extra volume in the weaker leg might improve ConPeak.

It is known that EOT has been implemented as a training strategy to minimize the risk of injury [38,39] in soccer players who are mostly involved in large eccentric force actions, such as changes of direction, accelerations, and decelerations [11]. Larger training effects were achieved in this study (ES: 1.00 to 1.97 in EccMean; ES: 0.77 to 1.63 in EccPeak) compared to previous studies (ES: 0.85 to 0.91 in EccMean; ES: 0.71 to 1.04 in EccPeak) [25,27]. In the same way as concentric variables, between-study differences in eccentric metrics might be due to the number of sessions carried out (10 vs. 6–7 sessions), the number of repetitions performed in each strategy (12–20 vs. 32–48 reps), or the type of exercises selected (unilateral vs. bilateral or combination of bilateral and unilateral EOT exercises). Thus, a lower volume of unilateral EOT exercises over a longer period might be a better training strategy to obtain improvements in eccentric power output. An interesting finding of the present study was the enhancement in the eccentric power output of the SVW group, obtaining better results than both DVW and SVS groups. Eccentric power CV values suggested poor reliability (17.48–24.87%); however, within-group changes were again much greater in all eccentric variables (EccMean stronger: 50.69–68.63%, EccMean weaker: 54.88–98.15%, EccPeak stronger: 54.19–74.98%, and EccPeak weaker: 63.18–90.92%). This, again, highlights where true change was evident, despite the larger-than-desired variability. In addition, between-groups comparison found no substantial differences between SVW and DVW. Hence, these results highlight the importance of starting with the weaker leg to achieve greater eccentric improvements.

Unilateral exercise seems to be an acceptable strategy for reducing inter-limb asymmetries [20,40]. Recent studies highlighted the variability of asymmetries across strength and jumping-based tests, suggesting that asymmetries are both test- and metric-specific [41]. As such, although other research studies showed a similar range of asymmetry reduction (ES: 0.01–0.88) [21,23,30], compared to the present study (ES: 0.01–1.60), different tests were performed, making direct comparisons challenging to make. In this regard, substantial effects were shown in asymmetry reduction in those groups that started performing the training program with the weaker leg. In particular, better improvements in concentric (ES: 0.89 in ConMean; ES: 1.60 in ConPeak) and EccMean (ES: 0.81) asymmetries were found in SVW, whereas ConMean (ES: 0.43) and EccPeak (ES: 0.68) asymmetry enhancements were found in DVW. Between-group results showed more benefits in decreasing asymmetries when training programmes started with the weaker leg. Another interesting finding is that, with the exception of EccPeak, favorable to DVW, no substantial differences were found between DVW and SVW for decreasing between-limb asymmetries. Therefore, this study suggests that beginning EOT with the weaker leg might also be a viable strategy for the reduction of between-limb asymmetries. This proposition goes in the same direction as another study, which obtained more benefits in asymmetry reduction in triple single horizontal jump in those groups that started their training intervention with the weaker leg [21].

There are some limitations to this study that should be acknowledged. Firstly, the inclusion of a control group would have been preferable in order to better elucidate the training effects in the intervention group. In addition, the influence of performing a number of repetitions in all the sets depending on the declination of a minimum target power might be noteworthy. Likewise, this would involve the monitorization of peak and mean power over all sessions, which may help us to understand the improvements reported in power performance and asymmetry reduction between sets and/or between sessions. Therefore, the comparison of different unilateral EOT interventions on concentric and eccentric peak and mean power and their corresponding effects on inter-limb asymmetries in other training subjects, with different age, gender, and sport, warrants further investigation.

## 5. Conclusions

Mean and peak power performance were substantially improved in all groups. A moderate reduction in mean and maximum peak concentric and eccentric asymmetry was achieved in those groups that started the program with the weaker leg. Finally, double volume starting with the weaker leg group developed better results in mean concentric power in comparison with the rest of groups.

Practitioners can directly include some specific guidelines to improve strength and power performance and to decrease inter-limb asymmetries. As team sports mainly depend on the application of force unilaterally, there is evidence to follow the principle of specificity: unilateral training should be included. Moreover, including an EOT might help inter-limbs asymmetries decrease, and this could reduce the risk of injury. Furthermore, to achieve the concurrent target of performance and injuries, strength unilateral training practices should be started with the weaker leg measured through the specific test or ability required on the pitch.

## Figures and Tables

**Table 1:** Familiarization, lateral squat eccentric overload training program, and testing sessions.

Week	Session	Sets/Leg	Repetitions/Set/Leg	Speed/Force Ratio
1	Familiarization			
2	Reliability test #1			
3	Reliability test #2			
4	Reliability test #3/Pre-test	2		1 out of 4
5–6	Session 1–2	2	6	1 out of 4
7–8	Session 3–4	2	8	1 out of 4
9–10	Session 5–6	2	8	2 out of 4
11–12	Session 7–8	2	10	2 out of 4
13–14	Session 9–10	2	10	3 out of 4
15	Post test	2		1 out of 4

**Table 2:** Measures of reliability in strength performance test (*n* = 45).

TEST	TEM (90% CL)	CV (90% CL)	ICC (90% CL)
ConMean stronger	71.45 (59.76; 89.51)	19.46 (16.03; 24.95)	0.75 (0.6; 0.85)
ConMean weaker	51.81 (43.34; 64.91)	16.86 (13.92; 21.56)	0.78 (0.64; 0.87)
EccMean stronger	76.9 (64.32; 96.33)	24.87 (20.41; 32.08)	0.66 (0.47; 0.79)
EccMean weaker	56.32 (47.11; 70.56)	18.97 (15.64; 24.31)	0.76 (0.61; 0.86)
ConPeak stronger	132.45 (110.78; 165.93)	18.88 (15.57; 24.2)	0.8 (0.67; 0.88)
ConPeak weaker	93.11 (77.88; 116.65)	16.28 (13.44; 20.79)	0.81 (0.68; 0.89)
EccPeak stronger	159.9 (133.74; 200.31)	18.14 (14.96; 23.23)	0.84 (0.73; 0.9)
EccPeak weaker	128.84 (107.76; 161.4)	17.48 (14.43; 22.36)	0.82 (0.7; 0.89)

Note. ConMean, mean concentric power output; EccMean, mean eccentric power output; ConPeak, maximal peak concentric power output; EccPeak, maximal peak eccentric power output; TEM, typical error of measurement; CL, confidence limits; CV, coefficient of variation expressed as percentage of TEM; ICC, intraclass correlation coefficient.

**Table 3:** Changes in Performance and Asymmetries After Unilateral Eccentric Overload Training With Different Strategies.

	SVW = SAME VOLUME, WEAKER LEG (*n* = 10)				DVW = DOUBLE VOLUME, WEAKER LEG (*n* = 11)				SVS = SAME VOLUME, STRONGER LEG (*n* = 14)			
Variables	PRE-TEST	POST-TEST	ES (CL90%)	*p*	PRE-TEST	POST-TEST	ES (CL90%)	*p*	PRE-TEST	POST-TEST	ES (CL90%)	*p*
ConMean stronger (W)	439.30 (±169.02)	747.22 (±203.79)	1.49 (1.1; 1.87)	<0.01	493.06 (±211.14)	807.29 (±200.33)	1.07 (0.64; 1.5)	<0.01	427.44 (±124.88)	648.45 (±178.59)	1.16 (0.81; 1.5)	<0.01
ConMean weaker (W)	306.91 (±202.13)	748.46 (±214.19)	2.24 (1.74; 2.74)	<0.01	358.72 (±211.22)	796.37 (±191.34)	2.04 (1.45; 2.64)	<0.01	339.79 (±138.56)	636.84 (±166.9)	1.53 (1.22; 1.85)	<0.01
ConMean asymmetry (%)	32.9 (±37.08)	7.84 (±5.38)	0.89 (0.19; 1.58)	0.04	32.56 (±36.35)	13.7 (±9.99)	0.43 (−0.15; 1.01)	0.18	20.74 (±24.85)	12.23 (±9.56)	0.45 (−0.31; 1.2)	0.54
EccMean stronger (W)	404.36 (±146.44)	669.52 (±176.23)	1.52 (1.15; 1.9)	<0.01	456.18 (±161.17)	705.89 (±165.03)	1.17 (0.71; 1.63)	<0.01	391.96 (±114.86)	577.28 (±154.35)	1.00 (0.71; 1.29)	<0.01
EccMean weaker (W)	344.28 (±138.17)	667.14 (±178.9)	1.97 (1.53; 2.4)	<0.01	443.38 (±165.13)	656.93 (±131.9)	1.08 (0.71; 1.45)	<0.01	367.91 (±114.39)	581.17 (±148)	1.16 (0.85; 1.47)	<0.01
EccMean asymmetry (%)	26.78 (±29.57)	11.43 (±6.36)	0.81 (−0.03; 1.65)	0.19	19.61 (±16.58)	13.65 (±6.23)	0.01 (−1.02; 1.04)	0.27	14.12 (±7.87)	9.37 (±9.25)	0.63 (−0.02; 1.28)	0.24
ConPeak stronger (W)	788.95 (±307.85)	1167.04 (±250.03)	1.14 (0.68; 1.61)	<0.01	916.66 (±434.15)	1348.97 (±322.06)	0.92 (0.43; 1.4)	<0.01	717.06 (±195.93)	1085.83 (±347.05)	1.25 (0.83; 1.67)	<0.01
ConPeak weaker (W)	669.49 (±313.97)	1217.9 (±302.24)	1.63 (1.13; 2.14)	<0.01	872.23 (±447.98)	1176.24 (±209.55)	0.77 (0.33; 1.21)	0.03	667.53 (±192.14)	1072.01 (±368.35)	1.33 (0.95; 1.71)	<0.01
ConPeak asymmetry (%)	32.02 (±28.36)	6.47 (±4.94)	1.6 (0.78; 2.41)	0.01	20.63 (±16.33)	16.43 (±11.28)	0.07 (−0.81; 0.94)	0.45	13.68 (±9.21)	10.49 (±8.98)	0.39 (−0.28; 1.06)	0.44
EccPeak stronger (W)	938.72 (±427.89)	1581.07 (±418.54)	1.31 (0.77; 1.86)	<0.01	1034.55 (±436.39)	1645.6 (±421.01)	1.12 (0.68; 1.57)	<0.01	850.57 (±297.64)	1311.17 (±466.67)	1.08 (0.71; 1.45)	<0.01
EccPeak weaker (W)	834.25 (±397.72)	1560.83 (±522.17)	1.64 (1.18; 2.09)	<0.01	1010.49 (±450.81)	1583.75 (±334.42)	1.11 (0.72; 1.5)	<0.01	812.37 (±311.59)	1311.07 (±451.86)	1.19 (0.83; 1.56)	<0.01
EccPeak asymmetry (%)	27.32 (±29.6)	14.36 (±11.17)	0.34 (−0.46; 1.13)	0.39	20.46 (±12.81)	12.2 (±10.4)	0.68 (0.09; 1.27)	0.11	13.32 (±11.39)	12.6 (±10.67)	0.08 (−0.66; 0.82)	0.89

Note: ConMean, mean concentric power output; EccMean, mean eccentric power; ConPeak, maximal peak concentric power output; EccPeak, maximal peak eccentric power output; ES, effect size; CL, confidence limit; SVW, unilateral eccentric overload training in the lateral squat performing the same volume with both limbs starting with the weaker limb; DVW, unilateral eccentric overload training in the lateral squat performing the double volume with the weaker limb starting with the weaker limb; SVS, unilateral eccentric overload training in the lateral squat performing the same volume with both limbs starting with the stronger limb. All results are presented in the same direction; that is, a positive change is considered an improvement, while a negative change is considered an impairment.

## Data Availability

Not applicable.
